# Mortality Variations of COVID-19 from Different Hospital Settings During Different Pandemic Phases: A Multicenter Retrospective Study

**DOI:** 10.5811/westjem.2021.5.52583

**Published:** 2021-09-02

**Authors:** Eric H. Chou, Chih-Hung Wang, Chu-Lin Tsai, John Garrett, Toral Bhakta, Andrew Shedd, Dahlia Hassani, Robert Risch, James d’Etienne, Gerald O. Ogola, Matthew Huei-Ming Ma, Tsung-Chien Lu, Hao Wang

**Affiliations:** * Baylor Scott and White All Saints Medical Center, Department of Emergency Medicine, Fort Worth, Texas; † John Peter Smith Hospital, Department of Emergency Medicine, Fort Worth, Texas; ‡ National Taiwan University Hospital, Department of Emergency Medicine, Taipei, Taiwan; § National Taiwan University College of Medicine, Department of Emergency Medicine, Taipei, Taiwan; ¶ Baylor University Medical Center, Department of Emergency Medicine, Dallas, Texas; || Baylor Scott and White Medical Center at Grapevine, Department of Emergency Medicine, Grapevine, Texas; # Baylor Scott & White Research Institute, Dallas, Texas

## Abstract

**Introduction:**

Diverse coronavirus disease 2019 (COVID-19) mortalities have been reported but focused on identifying susceptible patients at risk of more severe disease or death. This study aims to investigate the mortality variations of COVID-19 from different hospital settings during different pandemic phases.

**Methods:**

We retrospectively included adult (≥18 years) patients who visited emergency departments (ED) of five hospitals in the state of Texas and who were diagnosed with COVID-19 between March–November 2020. The included hospitals were dichotomized into urban and suburban based on their geographic location. The primary outcome was mortality that occurred either during hospital admission or within 30 days after the index ED visit. We used multivariable logistic regression to investigate the associations between independent variables and outcome. Generalized additive models were employed to explore the mortality variation during different pandemic phases.

**Results:**

A total of 1,788 adult patients who tested positive for COVID-19 were included in the study. The median patient age was 54.6 years, and 897 (50%) patients were male. Urban hospitals saw approximately 59.5% of the total patients. A total of 197 patients died after the index ED visit. The analysis indicated visits to the urban hospitals (odds ratio [OR] 2.14, 95% confidence interval [CI], 1.41, 3.23), from March to April (OR 2.04, 95% CI, 1.08, 3.86), and from August to November (OR 2.15, 95% CI, 1.37, 3.38) were positively associated with mortality.

**Conclusion:**

Visits to the urban hospitals were associated with a higher risk of mortality in patients with COVID-19 when compared to visits to the suburban hospitals. The mortality risk rebounded and showed significant difference between urban and suburban hospitals since August 2020. Optimal allocation of medical resources may be necessary to bridge this gap in the foreseeable future.

## INTRODUCTION

### Background

The coronavirus disease 2019 (COVID-19) continues its spread rapidly around the world. While patients with COVID-19 may manifest with minor symptoms, some may progress to critical illness, leading to severe disabilities or even death.^1^ Diverse COVID-19 mortalities have been reported from different studies among different patient populations during the beginning of this pandemic.^2^–^4^ Higher in-hospital mortalities have been reported among Black and Hispanic patients in the US.^5^ Nearly 50% of mortality was found among critically ill geriatric patients in Italy.^6^ Mortality doubled among patients who had certain comorbidities (eg, diabetes, obesity, cancer, and chronic renal insufficiency).^7^

These mortality differences were found at the individual patient level during the early COVID-19 pandemic phase, data that is helpful in identifying susceptible patients at risk of more severe disease or death. However, it does not provide enough information to determine mortality differences and its dynamical changes during the COVID-19 pandemic among different healthcare settings, which would be useful for overall COVID-19 resource reallocation.

### Importance

The surge in demand for hospital admissions and intensive care can quickly exceed the capacity of involved hospitals and deplete the available medical resources rapidly. The ability of each hospital to prioritize and mobilize its resources in response to medical needs may differ and may contribute to observed differences in mortality. However, certain changes can be dynamic at different pandemic phases. Determining different COVID-19 mortality patterns within different healthcare settings during different pandemic phases will help healthcare policymakers administer appropriate regulations to reasonably allocate medical resources, implement optimal care managements to flatten the surge waves, and minimize the mortality.

### Goals of This Investigation

In this study we aimed to investigate the mortality variations of patients with COVID-19 from different hospital settings during different pandemic phases in 2020. For the purpose of this study, we dichotomized the included hospitals into urban or suburban hospital based upon their geographic location.

## METHODS

### Study Design and Setting

Baylor Scott & White Health (BSWH) is the largest not-for-profit healthcare system in Texas, with 52 hospitals, more than 800 patient care sites, more than 7300 active physicians, and over 49,000 employees. This retrospective study was conducted by using data retrieved from the electronic health record (EHR) system of the five study hospitals affiliated with BSWH. Among these hospitals (Supplement Table S1), Baylor University Medical Center at Dallas (BUMC) and Baylor Scott & White All Saints Medical Center-Fort Worth (BAS) are categorized as urban hospitals, while Baylor Scott & White Medical Center-Grapevine (GRAP), Baylor Scott & White Medical Center-Irving (IRV), and Baylor Scott & White Medical Center-Waxahachie (WAX) are suburban hospitals. The treatment protocols did not vary between urban and suburban hospitals during the study period. This study was performed in accordance with the Declaration of Helsinki amendments. The institutional review board approved this study (reference number: 344143) and waived the requirement for informed consent because of the retrospective and non-interventional nature. The results are reported according to the Strengthening the Reporting of Observational Studies in Epidemiology (STROBE) guidelines.^8^

**Population Health Research Capsule**

What do we already know about this issue?

*Most of the published reports on coronavirus disease 2019 (COVID-19) focus on identifying susceptible patients at risk of more severe disease or death*.

What was the research question?

*Is there any mortality variation in patients with COVID-19 from different hospital settings during different pandemic phases?*

What was the major finding of the study?

*Visits to the urban hospital were associated with a higher risk of mortality in patients with COVID-19 when compared to visits to the suburban hospitals*.

How does this improve population health?

*Optimal reallocation of medical resources may be needed in locations where COVID-19 caseloads continue to increase*

### Selection of Participants

Patients who made their visits to the emergency departments (ED) of the study hospitals between March–November 2020 were screened. All adult (age ≥18 years) patients who were tested positive for severe acute respiratory syndrome coronavirus 2 (SARS-CoV-2) by quantitative reverse transcription polymerase chain reaction (RT-PCR) from samples collected through nasopharyngeal or oropharyngeal swabs during the index ED visit were included for analysis. If a single patient visited EDs of these study hospitals multiple times, only data of the first visit were extracted for analysis. We excluded patients with missing values of major variables (eg, mortality, or COVID-19 test results) from the final analyses. Because of limited capacity for quantitative RT-PCR testing during March and April 2020, COVID-19 screening was restricted to patients with contact or travel history or patients with suspicious laboratory or imaging findings. Since May 2020, the decision to have the RT-PCR test was left to the discretion of the ED attending physicians or advanced-practice providers who cared for the patient, without further limitations.

### Data Collection and Outcome Measures

We extracted clinical data from the health system’s electronic health record (Epic Systems Corporation, Verona, WI) with the use of an enterprise data warehouse. The following data were retrieved: demographic characteristics (age, gender, self-reported ethnic group, insurance plan, smoking history, and pregnancy status); comorbidities documented through diagnosis codes linked to ambulatory primary care and specialty encounters (asthma, coronary artery disease, cancer, congestive heart failure, cirrhosis, chronic kidney disease, chronic obstruction pulmonary disease, dementia, diabetes mellitus, hepatitis, human immunodeficiency virus status, hypertension, transplant); body mass index recorded within the previous 12 months prior to the index ED visit; visiting hospital; date and time of ED visits; presenting vital signs and acuity level recorded at ED triage; and whether chest radiograph or blood tests were performed during ED stay. Modified early warning score (MEWS) and national early warning score (NEWS) were computed according to the variables recorded at triage.^9^–^10^ Visits during night shifts were defined as patient visits occurring from 8 pm until 8 am the next day.

The primary outcome was all-cause mortality that occurred either during hospital admission or within 30 days after the index ED visit for patients with COVID-19. We checked the survival status of all included patients through hospital record on December 31, 2020, to ensure that all patients were followed up for at least one month.

### Primary Data Analysis

Categorical variables are presented as counts with proportions, and continuous variables are presented as medians with interquartile ranges. Categorical variables were examined by chi-square test while continuous variables were compared by Wilcoxon’s rank-sum test. A two-tailed *P*-value < 0.05 was considered significant. We calculated the odds ratio (OR) as the outcome measure. Multivariable logistic regression analyses were used to investigate the associations between variables of interest and outcomes. We placed all available independent variables in the regression model for selection, irrespective of whether they were considered as significant in univariate analyses. Generalized additive models (GAM) were used to explore non-linear effects of the continuous variables on outcomes and to identify the optimal cut-off points to transform these variables into categorical variables.^11^

We developed the final regression model by stepwise variable selection procedure with iterations between the forward and backward steps. Significance levels for entry were defined at 0.15 to avoid exclusion of potential variables. We determined the final regression model by excluding non-significant variables sequentially until all regression coefficients were significant. The interaction between hospital settings and different periods was assessed during the model-fitting process. We assessed the goodness of fit of the regression models by *c* statistics, the adjusted generalized *R*^2^ and the Hosmer-Lemeshow goodness-of-fit test. We entered and processed data with Excel 2019 (Microsoft Corporation, Redmond, WA) and analyzed the data with SPSS version 27 (IBM Corporation, Armonk, NY) or R 3.3.1 (R Foundation for Statistical Computing, Vienna, Austria).

## RESULTS

### Characteristics of Study Subjects

**Figure 1:**
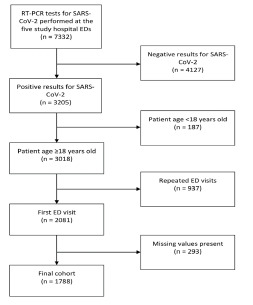
Patient inclusion flowchart. *RT-PCR*, reverse transcription polymerase chain reaction; *SARS-CoV-2*, severe acute respiratory syndrome coronavirus 2; *ED*, emergency department.

Between March–November 2020, a total of 7332 ED patient-visits were tested with RT-PCR for SARS-CoV-2 at the five study hospitals. Of them, 3018 adult (≥18 years) patient records with positive results were retrieved (Figure 1). After excluding 937 records due to repeated visits and 293 records with major missing variables, we included the remaining 1788 patient records in the study for analysis. The monthly ED volume from 2019 to 2020 stratified by the study hospitals are provided in Supplement Figure S1.

The features of the included patients are presented in Table 1. The median patient age was 54.6 years, and 897 patients (50.2%) were male. The most common comorbidity was hypertension (758, 42.4%), followed by diabetes mellitus (491, 27.5%). The proportions of patients with Medicaid/Medicare or commercial insurance were similar. Urban hospitals saw 59.5% of the total patients, and the majority of them made their visits to BUMC in this cohort. The number of COVID-19 patients reached its peak in July (828, 46.3%) and then gradually declined. The median body temperature and SpO_2_ measured at ED triage was 37.2°C and 96%, respectively. Approximately 34.2% of patients needed supplemental oxygen supplied at triage. The median MEWS and NEWS were 2 and 3, respectively. Most patients received a chest radiograph (1,355, 75.8%) and blood tests (1,568, 87.7%) during the index ED visit. A total of 197 patients (11.0%) died one month after the index ED visit or during the same admission after the index ED visits.

### Main Results

**Figure 2:**
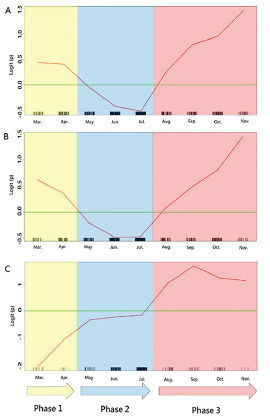
Generalized additive model plots for nonparametric modelling of the mortality variation (represented as logit of the probability of death) during different pandemic phases. A. Total cohort; B. Urban hospitals; C. Suburban hospitals. Logit (p), where p represented the probability for death.

The GAM plots illustrate the monthly variation effect on patient mortality, represented as logit (p), where p was the probability of death (Figure 2A). If logit (p) was greater than zero, the odds of mortality would be greater than one. The study period was thus divided into three phases: March–April defined as phase 1; May– July as phase 2; and August–November as phase 3 during the pandemic in 2020.

As shown in Table 2, the main analysis indicated that visits to the urban hospitals were positively associated with death (OR 2.14, 95% confidence interval [CI], 1.41, 3.23; *P*-value < 0.001). Also, compared with phase 2, visits made during phase 1 (OR 2.04, 95% CI, 1.08, 3.86; *P*-value = 0.03) and during phase 3 (OR, 2.15, 95% CI, 1.37, 3.38; *P*-value < 0.001) were also positively associated with death, respectively.

As shown in Figure 2B and 2C, the GAM plots revealed different mortality patterns for different hospital settings during different pandemic phases. For suburban hospitals, the mortality risk increased only during phase 3 and, therefore, only phase 3 was tested in the interaction analysis. A significant interaction was noted between the hospital settings and different pandemic phases. Compared with visits made to the suburban hospitals during phase 1 or phase 2, visits made to the urban hospitals during phase 1 had the highest mortality risk (OR, 4.48, 95% CI, 2.11, 9.50; *P*-value < 0.001), followed by visits made to the urban hospitals during phase 3 (OR 3.72, 95% CI, 2.13, 6.49; *P*-value < 0.001) (Table 3).

## DISCUSSION

### Main Findings

In the analysis we found that different hospital settings were significantly associated with mortality. That is, visits to the urban hospitals were associated with higher mortality, compared with visits to the suburban hospitals. We also noted a significant variation in mortality during different pandemic phases. The interaction analysis further revealed that urban hospitals were more sensitive to this mortality variation during different pandemic phases. As shown in Figure 2B, the mortality risk for urban hospitals during phase 3 (August–November 2020) rebounded as compared to the risk during phase 1 (March–April 2020). During phase 3, the risk of COVID-19 mortality was as high as 2.6-fold greater for urban hospitals, compared with suburban hospitals.

### Mortality Variation during Different Pandemic Phases

By using the GAM plots, our data revealed the mortality variation in COVID-19 as the pandemic was going on. As shown in Figure 2, there were two peaks in risk of mortality, ie, phase 1 and phase 3. The all-cause mortality during phase 1 was about 20% (23/118) in our study, similar to the mortality reported in New York City (21%) at the same time.^4^ The high mortality during this period was probably caused by the lack of understanding of a novel infectious disease, lack of well-equipped healthcare providers, and lack of proactive and prompt operational procedures in response to the pandemic. Furthermore, during phase 1, when the capacity for RT-PCR testing was limited in these study hospitals, it is likely that only those patients with clear contact or travel history or those with significant comorbidities received screening for COVID-19, resulting in a selection bias. Although some comorbidities might play important roles for mortality in this analysis, those unmeasured confounding factors may have led to falsely elevated mortality during phase 1.

Beginning in May 2020, the capacity for RT-PCR testing increased and the restrictions on testing decreased, leading to a surge in patients with a confirmed diagnosis of COVID-19. Despite this substantially increased patient number, the mortality during phase 2 was only 7% (99/1372), which is much lower than the mortality rate during phase 1. It might be argued that the substantial increase in patients with non-severe illness led to a relative decrease in mortality rate during phase 2. However, after the individual-level factors were considered in the analysis, patients presenting to EDs during phase 2 still had a lower risk of death. While the cause of this finding is likely multifactorial, one possibility is that hospitals experienced a reduction in care of other medical conditions, which increased their capacity to optimally care for patients with COVID-19.

As the pandemic proceeded, the mortality rate rose during phase 3 (August: 23%, September: 28%, October: 20%, November: 42%) despite the number of COVID-19 patients decreasing substantially. One possible explanation for this is that previously delayed medical care for other medical conditions had a negatively impactful rebound effect on the availability of resources for patients with COVID-19. As shown in Supplement Figure S1, the monthly total ED patient volumes increased from a nadir in April (about 50% of previous baseline) to a plateau after August (about 80% of previous baseline during phase 3). The competition between COVID-19 and other non-COVID-19 conditions for resources may also explain the mortality variation between urban and suburban hospitals.

### Mortality Variation from Different Hospital Settings

During phase 1, the initial epidemiologic data suggested that hospital mortality may not differ significantly across the United States.^12^–^14^ Nevertheless, a later multicenter study by Gupta et al indicated that one-month risk-adjusted mortality varied widely across 65 hospitals in the US, from 6.6% to 80.8%.^15^ Gupta et al identified substantial interhospital variation in the administration of medications and supportive therapies for treating COVID-19.^15^ This variation in clinical practice may have been caused by a lack of high-quality evidence in the optimal treatment during the initial period of the pandemic. For example, in the Gupta study, the proportion of patients who received hydroxychloroquine was 82.2% in average, with a range from 16.8% to 98.1%.^15^ Nonetheless, hydroxychloroquine was later found to be non-beneficial for hospitalized patients with COVID-19, and was not recommended in the latest treatment guidelines.^16^–^17^ Although there is no uniform recommendation for treating COVID-19, the treatment strategies may not be significantly different across the study hospitals given that regular meetings and discussion were held in our care system.

In our study, the mortality variation was primarily associated with different hospital settings. Visits to the urban hospitals were associated with higher mortality, compared with visits to the suburban hospitals. This difference became even more significant when patients made their visits to the urban hospitals during phase 1 and phase 3. For urban hospitals, the mortality risk during phase 3 was approaching that of phase 1 (Table 3) and seemed to have the potential to exceed it (Figure 2B). In contrast, for suburban hospitals, the mortality risk seemed to decrease (Figure 2C) after October. During phase 3, the risk of death in urban hospitals (OR 3.72) increased to as high as 2.6 times that of the suburban hospitals (OR 1.42) (Table 3).

One possible explanation may be that urban and suburban hospitals see patients with different socioeconomical backgrounds. Bambra et al reported significant variation in hospitalization rates and mortalities for COVID-19 across the New York City boroughs, with the highest rates of hospitalization and death happening to the borough with the highest proportion of racial/ethnic minorities and people living in poverty.^18^ Nevertheless, in our study we also took into account the influence of ethnicity and insurance plans, and found that patients of Hispanic ethnicity or patients with Medicaid/Medicare had higher risk of mortality after the infection of COVID-19. To some extent, the socioeconomic factors may be adjusted for in the regression analysis.

In a large cohort study, Asch et al found no association between academic status or urban/nonurban setting and a hospital’s mortality.^19^ Nonetheless, Asch et al included patients with COVID-19 between January–June 2020. During phase 3, because of the competing needs of other non-COVID-19 patients, the relative amount of resources dedicated to patients with COVID-19 may have decreased. As demonstrated in Supplement Figure S1, after August (ie, during phase 3), the monthly total ED patient volume increased to approximately 80% of baseline while the number of COVID-19 patients decreased (Table 1). This condition may be more pronounced in urban hospitals because they may have more non-COVID-19 patients to manage. For physicians in urban hospitals, because of the competing medical needs of both COVID-19 and non-COVID-19 patients, the increased workload and fatigue may have led to the substantial increase in COVID-19 mortality.^20^

### Future Applications

Our study indicates that urban hospitals have had a more challenging time dealing with COVID-19 patients during recent months (phase 3), compared with suburban hospitals. Despite the fact that COVID-19 vaccinations are currently available, it may take several months to achieve large-scale immunization and obtain herd immunity.^21^ Reallocation of medical resources may remain a necessary consideration to tide over this difficult interlude.

## LIMITATIONS

There were several limitations in this study. First, due to the retrospective nature of the study design, we could only establish an association, rather than a causal relationship, between the independent variables and outcomes. Second, the analyses were conducted based on data collected from a larger geographic location in Texas and may not be applicable to other population due to limited generalizability. Third, because not all the patients who visited the ED had laboratory or radiologic exams and not all of them were hospitalized, the influence of the exam results or course of hospitalization was unknown. Fourth, while we found mortality variations from different pandemic phases and different hospital settings in this study, we did not have detailed data to derive the exact mechanisms driving these variations.

## CONCLUSION

Patients with COVID-19 who visited urban hospital EDs had a higher mortality rate than patients who presented to suburban hospital EDs. The mortality rates initially decreased but then rebounded during recent months. In phase 3, the disparity in mortality between urban and suburban hospitals further increased and reached 2.6-fold. The consideration of optimal reallocation of medical resources may be necessary to bridge this gap for the foreseeable future in locations where COVID-19 caseloads continue to increase.

## Supplementary Information





## Figures and Tables

**Table 1:** Characteristics of patients with COVID-19 presenting to the emergency department.

Variables	Total (n=1,788)	Survival (n=1,591)	Death (n=197)	P value
Basic demographics				
Age, year	54.6 (41.9–68.2)	51.9 (40.5–65.6)	72.5 (64.5–80.3)	<0.001
Male	897 (50.2)	780 (49)	117 (59.4)	0.006
Body mass index, kg/m^2^	31.3 (27.0–37.0)	31.5 (27.2–37.2)	29.4 (25.8–35.9)	0.005
Hispanic ethnicity	908 (50.8)	823 (51.7)	85 (43.1)	0.02
Smoking history	368 (20.6)	307 (19.3)	61 (31.0)	<0.001
Pregnancy	44 (2.5)	44 (2.8)	0 (0)	0.02
Comorbidities				
Asthma	125 (7.0)	116 (7.3)	9 (4.6)	0.16
Cancer	57 (3.2)	41 (2.6)	16 (8.1)	<0.001
Chronic kidney disease	330 (18.5)	233 (14.6)	97 (49.2)	<0.001
Chronic obstructive pulmonary disease	196 (11.0)	155 (9.7)	41 (20.8)	<0.001
Cirrhosis	112 (6.3)	96 (6.0)	16 (8.1)	0.25
Congestive heart failure	210 (11.7)	146 (9.2)	64 (32.5)	<0.001
Coronary artery disease	197 (11.0)	147 (9.2)	50 (25.4)	<0.001
Dementia	116 (6.5)	89 (5.6)	27 (13.7)	<0.001
Diabetes mellitus	491 (27.5)	399 (25.1)	92 (46.7)	<0.001
Hepatitis	15 (0.8)	7 (0.4)	8 (4.1)	<0.001
Human immunodeficiency virus status	4 (0.2)	4 (0.3)	0 (0)	0.48
Hypertension	758 (42.4)	628 (39.5)	130 (66.0)	<0.001
Transplant	44 (2.5)	44 (2.8)	0 (0)	0.02
Insurance				<0.001
No insurance	352 (19.7)	340 (21.4)	12 (6.1)	
Medicaid/Medicare	716 (40.0)	553 (34.8)	163 (82.7)	
Commercial insurance	720 (40.3)	698 (43.9)	22 (11.2)	
Visiting hospital				<0.001
BUMC	699 (39.1)	591 (37.1)	108 (54.8)	
BAS	330 (18.5)	288 (18.1)	42 (21.3)	
GRAP	115 (6.4)	110 (6.9)	5 (2.5)	
IRV	334 (18.7)	315 (19.8)	19 (9.6)	
WAX	310 (17.3)	287 (18.0)	23 (11.7)	
Urban hospital	1,314 (59.5)	1,139 (57.5)	175 (77.1)	<0.001
Visit made at night shift	1,209 (67.6)	1,077 (67.7)	132 (67.0)	0.85
Monthly variation of visits				<0.001
March	49 (2.7)	43 (2.7)	6 (3.0)	
April	69 (3.9)	52 (3.3)	17 (8.6)	
May	158 (8.8)	145 (9.1)	13 (6.6)	
June	386 (21.6)	349 (21.9)	37 (18.8)	
July	828 (46.3)	779 (49.0)	49 (24.9)	
August	88 (4.9)	68 (4.3)	20 (10.2)	
September	61 (3.4)	44 (2.8)	17 (8.6)	
October	113 (6.3)	90 (5.7)	23 (11.7)	
November	36 (2.0)	21 (1.3)	15 (7.6)	
Vital signs at ED triage				
Temperature, °C	37.2 (36.8–37.9)	37.2 (36.9–37.9)	37.2 (36.8–38.1)	0.58
Heart rate, beats per minute	96 (84–110)	96 (84–109)	96 (85–112)	0.20
Respiratory rate, breaths per minute	20 (18–24)	20 (18–23)	22 (22–27)	<0.001
Mean blood pressure, mm Hg	96 (87–106)	96 (87–106)	93 (81–104)	0.001
SpO_2_, %	96 (92–98)	96 (93–98)	92 (83–96)	<0.001
Glasgow Coma Scale	15 (15–15)	15 (15–15)	15 (14–15)	<0.001
Triage acuity				<0.001
Level 1	87 (4.9)	58 (3.6)	29 (14.7)	
Level 2	785 (43.9)	660 (41.5)	125 (63.5)	
Level 3	828 (46.3)	785 (49.3)	43 (21.8)	
Level 4	81 (4.5)	81 (5.1)	0 (0)	
Level 5	7 (0.4)	7 (0.4)	0 (0)	
Supplemental oxygen supplied at ED triage	611 (34.2)	508 (31.9)	103 (52.3)	<0.001
MEWS	2 (1–4)	2 (1–3)	3 (2–5)	<0.001
NEWS	3 (2–6)	3 (1–6)	6 (4–9)	<0.001
CXR exam at ED	1,355 (75.8)	1,180 (74.2)	175 (88.8)	<0.001
Blood test at ED	1,568 (87.7)	1, 372 (86.2)	196 (99.5)	<0.001

Data are presented as median (interquartile range) or counts (proportion).

BAS, Baylor Scott & White All Saints Medical Center - Fort Worth; BUMC, Baylor University Medical Center at Dallas; ED, emergency department; GRAP, Baylor Scott & White Medical Center – Grapevine; IRV, Baylor Scott & White Medical Center – Irving; WAX, Baylor Scott & White Medical Center – Waxahachie.

*ED*, emergency department; *C*, Celsius; *SpO_2_*, oxygen saturation; *MEWS*, modified early warning score; *NEWS*, national early warning score; *CXR*, chest radiograph.

**Table 2:** Multivariable logistic regression model with death as the dependent variable.

Independent variable	Odds ratio	95% confidence interval	P value
Age (per year)	1.07	1.05–1.09	<0.001
NEWS	1.26	1.18–1.34	<0.001
Chronic kidney disease	2.11	1.42–3.14	<0.001
Urban hospital	2.14	1.41–3.23	<0.001
Visit made during phase 3	2.15	1.37–3.38	<0.001
Hispanic ethnicity	1.91	1.29–2.83	0.001
Medicaid/Medicare	2.22	1.30–3.78	0.003
Congestive heart failure	1.92	1.24–2.97	0.003
Respiratory rate >16 or <25	1.91	1.25–2.92	0.003
Glasgow Coma Scale (per unit increase)	0.89	0.82–0.96	0.004
CXR exam at ED	2.07	1.19–3.62	0.01
Transplant	2.91	1.08–7.85	0.03
Hepatitis	4.41	1.16–16.82	0.03
Visit made during phase 1	2.04	1.08–3.86	0.03
Body mass index >28 (kg/m^2^)	1.52	1.02–2.26	0.04
Dementia	0.53	0.29–0.96	0.04

Goodness-of-fit assessment: n = 1,788, adjusted generalized R^2^ = 0.44, estimated area under the receiver operating characteristic curve = 0.90, and Hosmer-Lemeshow goodness-of-fit chi-squared test P = 0.61.

The display of independent variables is arranged in order of p value.

CXR, chest radiograph; *ED*, emergency department; *NEWS*, national early warning score.

**Table 3:** Interaction analysis between hospital level and different phases during the pandemic.

Independent variable	Odds ratio	95% confidence interval	P value
Suburban hospital × Visits made at phase 1 or phase 2	Reference		
Suburban hospital × Visits made at phase 3	1.42	0.67–3.04	0.36
Urban hospital × Visits made at phase 2	1.77	1.06–2.93	0.03
Urban hospital × Visits made at phase 3	3.72	2.13–6.49	<0.001
Urban hospital × Visits made at phase 1	4.48	2.11–9.50	<0.001

Other variables adjusted in the model include: age, chronic kidney disease, congestive heart failure, chest radiograph exam at emergency department, dementia, Glasgow coma scale, hepatitis, Hispanic ethnicity, Medicaid/Medicare, national early warning score, respiratory rate, SpO_2_, transplant.

Goodness-of-fit assessment: n = 1,788, adjusted generalized R^2^ = 0.44, estimated area under the receiver operating characteristic curve = 0.90, and Hosmer and Lemeshow goodness-of-fit Chi-Squared test p = 0.61.
