# Supplementary material for: Mortality Variations of COVID-19 from Different Hospital Settings During Different Pandemic Phases: A Multicenter Retrospective Study
Source: West J Emerg Med. 2021 Sep 2;22(5):1051–9. doi: 10.5811/westjem.2021.5.52583 (PMC8463069; doi:10.5811/westjem.2021.5.52583)
Supplement: Supplementary file 1 [file wjem-22-1051-s001.docx]

**Supplemental Figure S1.** Line graph of the monthly patient volume visiting emergency departments of the study hospitals.


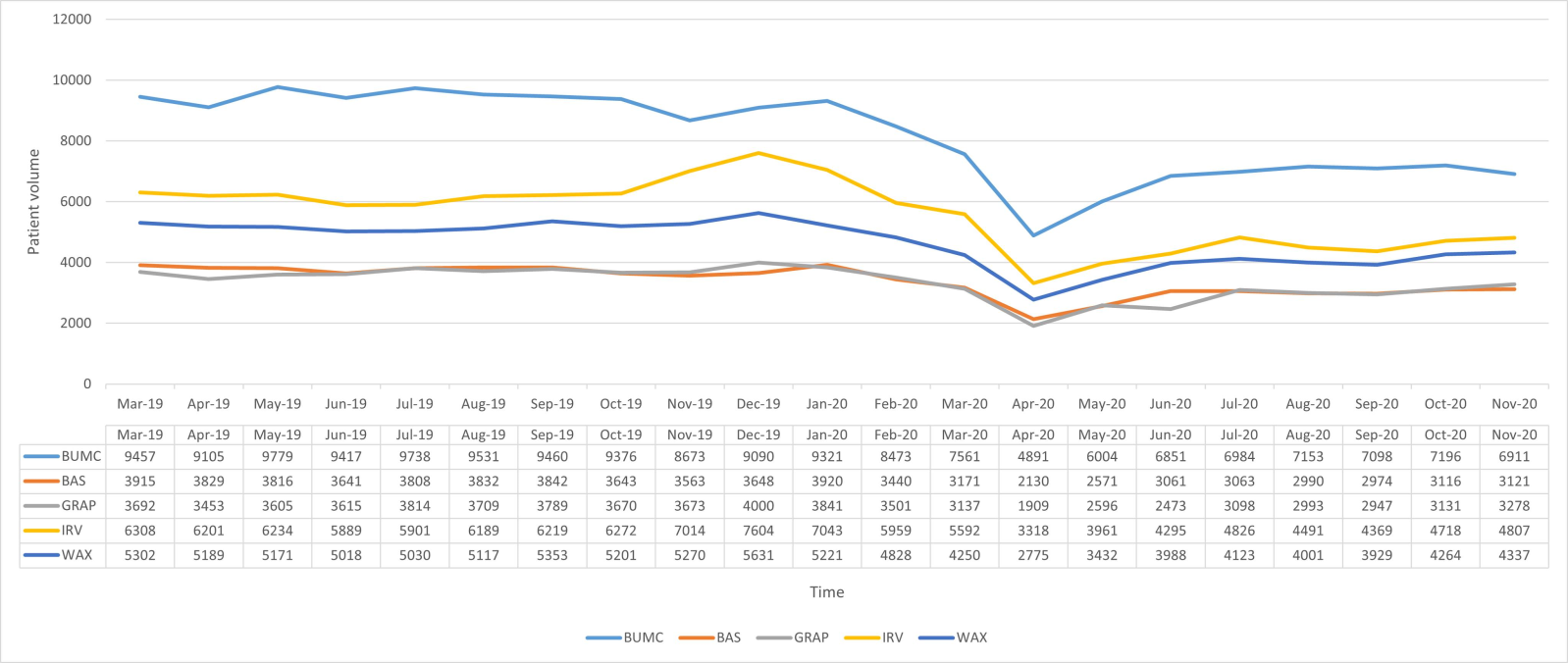


IRV: Baylor Scott & White Medical Center – Irving, WAX: Baylor Scott & White Medical Center – Waxahachie.BUMC: Baylor University Medical Center at Dallas, BAS: Baylor Scott & White All Saints Medical Center - Fort Worth, GRAP: Baylor Scott & White Medical Center – Grapevine.
