# Supplementary material for: Mortality Variations of COVID-19 from Different Hospital Settings During Different Pandemic Phases: A Multicenter Retrospective Study
Source: West J Emerg Med. 2021 Sep 2;22(5):1051–9. doi: 10.5811/westjem.2021.5.52583 (PMC8463069; doi:10.5811/westjem.2021.5.52583)
Supplement: Supplementary file 2 [file wjem-22-1051-s002.docx]

| Variables | BUMC | BAS | GRAP | IRV | WAX |
| --- | --- | --- | --- | --- | --- |
| Type | Urban, tertiary care, teaching hospital | Urban, tertiary care, teaching hospital | Suburban, community hospital | Suburban, community hospital | Suburban, community hospital |
| Hospital bed number | 1079 | 530 | 302 | 296 | 120 |
| ICU bed number | 128 | 42 | 42 | 49 | 12 |
| Annual ED volume | 90000 | 40000 | 40000 | 60000 | 50000 |
| Consultation services | All surgery/medical subspecialties available | All surgery/medical subspecialties available except Ophthalmology | Basic medical/surgical services including cardiovascular, neurology, and critical care services | Basic medical/surgical services including cardiovascular, neurology, and critical care services | Basic medical/surgical services including cardiovascular and critical care services |
| ED admission rate (%) | 28.0 | 32.9 | 29.3 | 16.7 | 15.2 |
| ED mortality rate (%) | 0.44 | 0.12 | 0.2 | 0.14 | 0.18 |
| ED boarding time (minute) | 46.7 | 42.0 | 39.5 | 25.9 | 30.2 |
| Door-to-provider time (minute) | 10.1 | 13.7 | 6.6 | 10.2 | 17.8 |
| ED acuity (%) |  |  |  |  |  |
| Level 1 | 1.29 | 0.41 | 1.88 | 0.8 | 2.66 |
| Level 2 | 22.61 | 18.84 | 27.88 | 19.81 | 20.64 |
| Level 3 | 52.45 | 64.29 | 52.15 | 49.66 | 52.15 |
| Level 4 | 19.26 | 14.87 | 15.55 | 23.58 | 22.56 |
| Level 5 | 3.97 | 1.07 | 1.55 | 4.41 | 1.27 |

**Supplemental Table**
